# Characterization of the *Akirin* Gene and Its Role in the NF-κB Signaling Pathway of *Sogatella furcifera*

**DOI:** 10.3389/fphys.2018.01411

**Published:** 2018-10-08

**Authors:** Jing Chen, Dao-Wei Zhang, Xing Jin, Xian-Lin Xu, Bo-Ping Zeng

**Affiliations:** ^1^Department of Biochemistry and Molecular Biology, College of Basic Medical Science, Zunyi Medical University, Zunyi, China; ^2^School of Biological and Agricultural Science and Technology, Zunyi Normal University, Zunyi, China

**Keywords:** *Sogatella furcifera*, Akirin, NF-κB signaling pathway, RNA interference, bacterial challenge

## Abstract

Akirin is an essential nuclear protein involved in the regulation of NF-κB signaling pathway. In most invertebrates, Akirin regulates NF-κB-related Imd and Toll pathways, however, in *Drosophila*, it only controls the Imd pathway, whereas its role in NF-κB signaling pathway in other insect species is unclear. In the present study, we used white-backed planthopper *Sogatella furcifera* as a model to investigate the functional activity of Akirin in insects. The sequence of *Akirin* cDNA was extracted from transcriptome database of *S. furcifera*; it contained a 585 bp open reading frame (ORF) encoding a putative protein of 194 amino acids. *S. furcifera* Akirin (SfAkirin) had a molecular weight of about 21.69 kDa and a theoretical pI of 8.66 and included a nuclear localization signal (NLS) of five amino acid residues at the N-terminal region. Evolutionary analysis showed that SfAkirin was evolutionary closer to Akirins of such relatively distant species as crustaceans than to those of some insect orders like Diptera and Hymenoptera. Tissue-specific expression analysis showed that the *SfAkirin* gene was expressed in all examined tissues, with the highest expression levels detected in the testis, followed by the ovary, whereas the lowest expression was found in the head. Real-time quantitative PCR analysis showed that *SfAkirin* mRNA was strongly induced in response to injection of heat-inactivated *Escherichia coli* and *Bacillus subtilis*, whereas *SfAkirin* silencing by RNA interference significantly reduced the expression of NF-κB dependent transcription factors *Dorsal* and *Relish* after *B. subtilis* and *E. coli* challenge, respectively. Our results suggest that SfAkirin may control the immune response of *S. furcifera* against bacterial infection via both Imd and Toll signaling pathways.

## Introduction

NF-κB is a nuclear transcription factor that activates the expression of genes involved in the immune response of insects to infections by pathogenic microorganisms and, therefore, plays a central role in the insect innate immunity (Hoffmann, 2003; Hultmark, 2003; Imler and Bulet, 2005). There are three types of NF-κB transcription factors identified in insects: Dorsal, Dif, and Relish (Eleftherianos et al., 2013; Zhong et al., 2016), which participate in immunity-related Toll and Imd signaling pathways (Lemaitre et al., 1995; Valanne et al., 2011; Myllymaki et al., 2014). Dorsal and Dif are the downstream transcription factors involved in the Toll pathway which primarily regulates the immune response to fungi and gram-positive bacteria (Valanne et al., 2011), whereas Relish acts in the Imd pathway which mainly controls the response to gram-negative bacteria (Georgel et al., 2001). In *Drosophila melanogaster, Bombyx mori*, and mosquitoes, the classical pathways through which NF-κB transcription factors participate in Toll and Imd signaling pathway are relatively well studied and described (Zou et al., 2011; Kuo et al., 2012; Hua et al., 2016; Salminen and Ramet, 2016). Nevertheless, an increasing number of new studies continue to contribute important data, furthering our understanding of these pathways and the related mechanisms. Wang et al. (2012) identified a novel transcription factor, lipopolysaccharide (LPS)-induced TNF-α factor (LITAF), which plays a role distinct from that of NF-κB in the Toll and Imd signaling pathways. In another study, Shi et al. (2012) demonstrated a possibility of different pairing of Toll with a cytokine-like peptide Spaztle and suggested functional roles of multiple Toll signal transduction pathways in insect immunity. The authors also showed, for the first time, that the insect myeloid differentiation factor 2 (MD-2) could have a function similar to that of the mammalian MD-2 in immune signaling and expression control of antimicrobial peptides cathelicidins and proposed a Toll-LPS signaling pathway regulating immunity in insects.

The *Akirin* gene was discovered by Goto et al. (2008) through whole-genome RNA interference (RNAi) screening in *Drosophila*. Akirin is strictly localized in the nucleus where it acts as a critical transcription factor regulating the innate immune response by controlling the expression of genes involved in the NF-κB-mediated Imd signaling pathway (Ferrandon et al., 2007; Goto et al., 2008; Bonnay et al., 2014). Previous studies have indicated that the *Akirin* gene exists only in animals and is not found in plants, fungi, or bacteria (Carreon et al., 2012; Manzano-Roman et al., 2012; Carpio et al., 2013; Hou et al., 2013; Moreno-Cid et al., 2013). It has been reported that the Akirin protein plays a role in transcriptional regulation of genes related to the NF-κB signaling pathway both in vertebrates such as mice and invertebrates such as *D. melanogaster* (insect) (Goto et al., 2008), *Litopenaeus vannamei* and *Caligus rogercresseyi* (crustaceans) (Carpio et al., 2013), and *Ixodes scapularis* (arachnid) (Naranjo et al., 2013). In *Drosophila*, Akirin downregulation can result in a major decrease in the expression of the Imd pathway genes *Imd, Relish*, and *PGRP-LC*, as well as an Imd target gene *Diptericin*, which corresponds to a significant increase in *Drosophila* sensitivity to gram-negative bacteria, as evidenced by the elevation of the mortality rate up to 100% at 24 h after bacterial infection. However, target genes of the Toll pathway, such as *Toll* and *Drosomycin*, are not affected by Akirin downregulation. These results clearly indicated that in *Drosophila*, Akirin plays a key role in the regulation of the Imd but not Toll signaling pathway (Goto et al., 2008; Bonnay et al., 2014), which is in contrast to its function in other invertebrates such as *C. rogercresseyi* and *L. vannamei*, where it controls both Imd and Toll pathways (Carpio et al., 2013; Hou et al., 2013; Naranjo et al., 2013). In addition to its involvement in the regulation of NF-κB-mediated signaling, Akirin takes part in biological processes unrelated to immunity. For example, *Akirin* gene knockdown by RNA interference in mosquitoes suggested a role for Akirin in mosquito survival and fertility. Vaccination of mice with recombinant *Aedes albopictus* Akirin reduced parasite infection in mosquitoes fed on immunized mice compared to controls (da Costa et al., 2014). RNAi-mediated silencing of *Akirin* gene expression has shown to protect against tick infestations by reducing tick fertility and vectorial capacity (de la Fuente et al., 2006, 2008, 2011; Harrington et al., 2009; Moreno-Cid et al., 2010). In *Xenopus*, Akirin was found to exert precise temporal control over *Xenopus* neural development (Liu et al., 2017). In mammals, Akirin functions as an important myogenic factor negatively regulated by myostatin, affecting biological processes such as mouse embryonic development and postnatal muscle growth, as well as intramyocellular lipid content (Sasaki et al., 2009). Akirin down-regulation increases chemosensitivity in human glioblastomas more efficiently than that by Twist-1 down-regulation (Krossa et al., 2015). Moreover, Akirin is considered to be a promising target for the development of vaccines to control mosquitoes, hard ticks, and sand flies (de la Fuente et al., 2006, 2011; Galindo et al., 2009; Harrington et al., 2009; Prudencio et al., 2010; Merino et al., 2011; Carreon et al., 2012; Manzano-Roman et al., 2012; Carpio et al., 2013).

The white–backed planthopper *S. furcifera* belonging to the Hemiptera order is an insect with incomplete metamorphosis, which has become one of the most destructive pests in rice production in Asia. *S. furcifera* can migrate over long distances in temperate and tropical regions of Asia and primarily feeds on rice plants (Shen et al., 2005; Ma et al., 2017), from which it sucks sap, causing yellowing, stunting, and hopper burn, and finally plant death (Wang et al., 2017). More importantly, *S. furcifera* can transmit dangerous rice viruses, further contributing to the damage of rice plants (Zhou et al., 2013; He et al., 2016; Wu et al., 2017). Therefore, it is important to investigate the immune mechanisms of *S. furcifera* to develop effective control strategies. Among insects, Akirin regulation of the NF-κB signaling has only been reported in *Drosophila*, but not in other species. It has been shown that in *Drosophila*, Akirin is involved in the regulation of the Imd but not Toll pathway. The main objective of the present study was to investigate the association between Akirin and NF-κB signaling in *S. furcifera* in order to determine whether a universal regulation system similar to that in *Drosophila* exists in all insects or there are species-specific differences. The results of the present study should advance the knowledge of the mechanisms underlying the role of the NF-κB signaling pathway in the innate immunity of *S. furcifera* in particular and insects in general.

## Materials and Methods

### Insects

*Sogatella furcifera* used in this study was obtained from the Zhejiang University (Hangzhou, China). The insects were maintained on rice (variety Taichung Native 1 [TN1]) for more than 30 generations under following conditions: temperature, 27 ± 0.5°C; relative humidity, 70 ± 5%; photoperiod, 16:8 h (light:dark). TN1 seedlings were grown in soil at 28–30°C under a long photoperiod (14:10 h light:dark) in an artificial-climate room and grasshoppers were transferred to fresh seedlings every 12–15 days to assure sufficient nutrition.

### Sequence and Phylogenetic Analysis of *SfAkirin*

The sequence of the *SfAkirin* unigene was obtained from the corresponding *S. furcifera* transcriptome datasets recently constructed in our laboratory. The open reading frame (ORF) was identified using the EditSeq program of DNAStar and molecular weight and theoretical pI of the SfAkirin protein were deduced using the ExPASy-ProtParam tool^1^. Homologous genes from other insect species were identified by similarity search using NCBI-BLAST^2^, and multiple sequence alignments were performed with Clustalx 1.83. A phylogenetic tree was constructed with MEGA 7.0 using the Neighbor-joining method. The following Akirins were used in multiple sequence alignment and phylogenetic analysis: AmAkirin (*Apis mellifera*, XP_395252.2), ObAkirin (*Ooceraea biroi*, EZA59122.1), SiAkirin (*Solenopsis invicta*, XP_011169583.1), AeAkirin (*Acromyrmex echinatior*, XP_011066641.1), SfAkirin (*S. furcifera*, MG744348), ApAkirin (*Acyrthosiphon pisum*, XP_001943118), RpAkirin (*Riptortus pedestris*, BAN21089), AaAkirin (*A*. *albopictus*, ACF49499.1), AgAkirin (*Anopheles gambiae*, XP_308938.4), CqAkirin (*Culex quinquefasciatus*, XP_001863200.1), DmAkirin (*D*. *melanogaster*, NP_648113.1), BmAkirin (*B*. *mori*, NP_001243977.1), PaAkirin (*Pararge aegeria*, JAA80553.1), and akirins of *Linepithema humile* (XP_012216183.1), *Trachymyrmex septentrionalis* (KYN34212.1), *Nasonia vitripennis* (XP_008215008.1), *Eriocheir sinensis* (AGT21376.1), *L*. *vannamei* (AGG35614.1), *Centruroides sculpturatus* (XP_023221383.1), *Ornithodoros moubata* (AGI44628.1), *I*. *scapularis* (AGO59321.1), *Haemaphysalis elliptica* (AGI44626.1), *Rhipicephalus microplus* (AFH57345.1), *Musca domestica* (XP_005185646.1), *Bactrocera oleae* (NP_001302560.1), *Ceratitis capitata* (XP_004526166.1), *Nicrophorus vespilloides* (XP_017785343), *Tribolium castaneum* (XP_971340), *Bos taurus* (Akirin-1, NP_001094706.1, and Akirin-2, NP_001103557.1), *Ovis aries* (Akirin-1, NP_001121144.1, and Akirin-2, NP_001239105.1), *Sus scrofa* (Akirin-1, AIK19308.1, and Akirin-2, AGA94528.1), *Homo sapiens* (Akirin-1, NP_078871.1, and Akirin-2, NP_060534.1), and *Mus musculus* (Akirin-1, NP_075912.2, and Akirin-2, NP_001007590.2).

### Tissue Distribution of *SfAkirin* mRNA

Total RNA was extracted from different tissues of individual fifth-instar *S. furcifera* nymphs, including the head, fat body, gut, wind, cuticle, muscle, testis, and ovary using an RNA extraction kit (Tiangen Biotech Co, China), and 2 μg RNA was used to synthesize first-strand cDNA with the one step SYBR PrimerScript RT-PCR Kit (Takara, Japan). Real-time quantitative PCR (qRT-PCR) was used to quantify *SfAkirin* expression in different *S. furcifera* tissues with the actin 1-encoding gene (GenBank ID: ALO78726.1) as an internal reference control; primers (Sangon Biotech, China) are shown in **Table 1**. All qRT-PCR reactions were performed in triplicate in a Bio-Rad Real-Time PCR Detection System (BioRad, Hercules, CAı, United States) at the following conditions: initial denaturation at 95°C for 10 s, followed by 40 cycles of denaturation at 95°C for 5 s and annealing/extension at 60°C for 30 s. The relative gene expression of *SfAkirin* was calculated using the comparative 2^-ΔΔCT^ Ct method (ΔΔCT = ΔCT _target_ - ΔCT _reference_).

**Table 1:** Primers used in this study.

Primer use	Primer names	Nucleotide sequences (5′→3′)
Amplification of *Akirin* dsRNA	Akirin-dsRNA-F	CGTAAGCAGTTGCACTTTAATCC
	Akirin-dsRNA-R	GCTGGTCGTTGGAGAACTTG
	Akirin-dsRNA-T7F	GGATCCTAATACGACTCACTATAGGCGTAAGCAGTTGCACTTTAATCC
	Akirin-dsRNA-T7R	GGATCCTAATACGACTCACTATAGGGCTGGTCGTTGGAGAACTTG
Amplification of *GFP* dsRNA	GFP-dsRNA-F	AAGGGCGAGGAGCTGTTCACCG
	GFP-dsRNA-R	CTTGACCTCGGCACGCGTCTTGT
	GFP-dsRNA-T7F	GGATCCTAATACGACTCACTATAGGAAGGGCGAGGAGCTGTTCACCG
	GFP-dsRNA-T7R	GGATCCTAATACGACTCACTATAGGCTTGACCTCGGCACGCGTCTTGT
qRT-PCR for *Akirin*	Realtime-Akirin-F	GTTCTCCACCTACAACTTCTACAA
	Realtime-Akirin-R	GCGAATCTCCTCACGAATACC
qRT-PCR for *Actin 1*	Realtime-Actin-F	CGTCTACAACTCCATCATGAAGTG
	Realtime-Actin-R	ATGATCTTGATCTTGATGGTTGAGG
qRT-PCR for *Dorsal*	Realtime-Dorsal-F	CGGCTACGAACACAAGAACCA
	Realtime-Dorsal-R	GTAGATAGGCTCCGATACGACTG
qRT-PCR for *Relish*	Realtime-Relish-F	TGTGAACCTGTCTACTCTCAACCT
	Realtime-Relish-R	ATATCCTCTCCTCCTTCGCATGA

### Bacterial Induction

Gram-negative *E. coli* strain K12 and gram-positive *B. subtilis* were used to inject *S. furcifera*. Bacteria were cultured overnight at 37°C on LB agar plates using the streak-plate procedure. A single colony of *E. coli* or *B. subtilis* was used to inoculate 10 ml of LB broth in culture flasks, which were then incubated at 37°C with shaking at 200 rpm until optical density at 600 nm reached 0.6 and 0.75, respectively. The bacteria were then collected by centrifugation at 5,000 rpm for 5 min, resuspended in phosphate-buffered saline (PBS, 137 mM NaCl, 2.68 mM KCl, 8.1 mM Na_2_HPO_4_, 1.47 mM KH_2_PO_4_, pH 7.4) to a density of 10^6^ cells/ml, and heat-killed by boiling for 30 min.

Fourth-instar *S. furcifera* nymphs (day 1) were evenly distributed into three injection groups: *E. coli, B. subtilis*, and PBS (control). The insects were anesthetized with CO_2_ (pressure: 5 mPa) for 10 s and fixed on an agarose gel-casting tray in a neat array with their abdomens facing upwards. Then, 0.5 μl of inactivated bacteria or PBS was injected into the abdomen segment junction between the second and third appendages using a FemtoJet microinjection system (Eppendorf, Germany). The injected nymphs were reared in the incubation chamber, fed with fresh rice, and analyzed for *Akirin* gene expression by qRT-PCR as described.

### RNA Interference

To synthesize dsRNA, a 310 bp fragment of *SfAkirin* and a 333 bp fragment of GFP-encoding gene (GenBank ID: KU306402.1) were amplified by PCR using the *S. furcifera* cDNA and plasmid pJV53-GFP, respectively. Akirin dsRNA was synthesized with primers Akirin-dsRNA-F, Akirin-dsRNA-T7F, Akirin-dsRNA-R, and Akirin-dsRNA-T7R, and GFP dsRNA was synthesized with primers GFP-dsRNA-F, GFP-dsRNA-T7F, GFP-dsRNA-R, and GFP-dsRNA-T7R (**Table 1**) using the T7 RiboMAX^TM^ Express RNAi System (Promega, Madison, WI, United States). The reactions were performed at the following conditions: 95°C for 5 min followed by 35 cycles at 95°C for 30 s, 55°C for 30 s and 72°C for 30 s, and a final extension at 72°C for 10 min. The resultant dsRNA products were washed with 70% ethanol, dried, re-suspended in nuclease-free water, and quantified using a BioSpectrometer (Eppendorf, Germany). The quality and size of dsRNA were checked by electrophoresis in 1.5% agarose gels.

In the dsRNA injection experiment, we used the rearing procedure described earlier (Waris et al., 2018). Briefly, fourth-instar *S. furcifera* nymphs (day 1) were equally divided into the dsAkirin injection group and dsGFP control group, anesthetized and fixed as described above, and injected with 10 ng Akirin dsRNA or GFP dsRNA, respectively, into the abdomen segment junction between the second and third appendages using the FemtoJet microinjection system. RNA was extracted from nine individual insects per group at 24 and 48 h post-injection, and RNA samples from three insects in each group were combined and used for one reaction.

### Effect of *SfAkirin* on *Dorsal* and *Relish* Expression *in vivo*

To determine whether SfAkirin participates in the activation of the Toll and Imd pathways after bacterial infection, *Dorsal* (GenBank ID: AWT86616) mRNA expression was determined in dsAkirin-injected *S. furcifera* challenged or not with *E. coli* (dsAkirin + *E. coli*) or *B. subtilis* (dsAkirin + *B. subtilis*) and *Relish* (GenBank ID: AWT86617) mRNA expression was determined in the dsAkirin + *E. coli* group; dsGFP-injected *S. furcifera* challenged or not with *E. coli* (dsGFP + *E. coli*) or *B. subtilis* (dsGFP + *B. subtilis*) was used as control. *S. furcifera* nymphs were injected with dsRNA and challenged with bacteria 24 h later; *Dorsal* and *Relish* mRNA levels were determined at 24, 48, and 72 h after bacterial challenge by qRT-PCR using primers Realtime-Dorsal-F and Realtime-Dorsal-R, and Realtime-Relish-F and Realtime-Relish-F, respectively (**Table 1**).

### Statistical Analysis

Real-time quantitative PCR statistical analysis was performed by one-way Analysis of Variance (ANOVA) and Student’s *t*-test using the SPSS 19.0 software (SPSS, Chicago, IL, United States) to compare mRNA expression in different groups. Differences at *P* < 0.05 were considered statistically significant.

## Results

### *Akiri*n Gene Sequence Analysis

**FIGURE 1:**
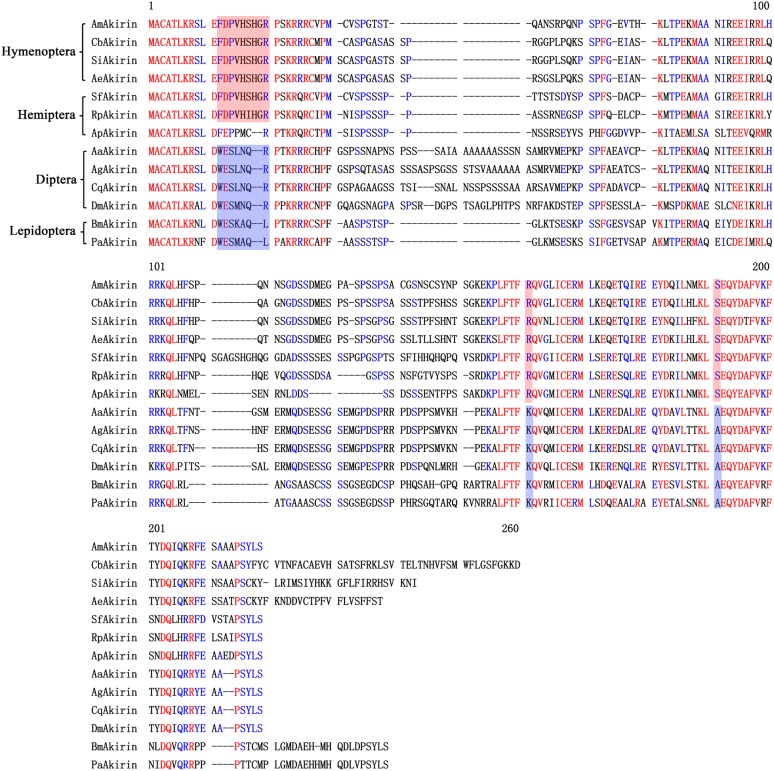
Alignment of the amino acid sequence of SfAkirin with those of other insect Akirins. A conserved nuclear localization signal (NLS) is shaded gray; identical sequences in conserved regions of Hymenoptera and Hemiptera species are shaded red and those for Diptera and Lepidoptera species are shaded blue.

The cDNA of *SfAkirin* (GenBank ID: AVW83290.1) contains an ORF of 585 bp, which encodes a putative protein of 194 amino acids. The predicted molecular weight of SfAkirin is about 21.69 kDa with a theoretical pI of 8.66. Homology analysis of insect Akirins using the clustalx software indicated that a high degree of homology (about 50–60%) existed among these proteins in insects from different orders (**Figure 1**). Furthermore, highly conserved amino acid motifs were found in various SfAkirin regions, including the KRR/QRC sequence at the N-terminus (residues 23–27), which was identified as a nuclear localization signal (NLS) (**Figure 1**). The results of multiple sequence alignment among Akirins of 13 insects from orders Hymenoptera, Hemiptera, Diptera, and Lepidoptera revealed conserved sequences at the extreme N-termini and C-termini (**Figure 1**).

### Evolutionary Analysis of SfAkirin

**FIGURE 2:**
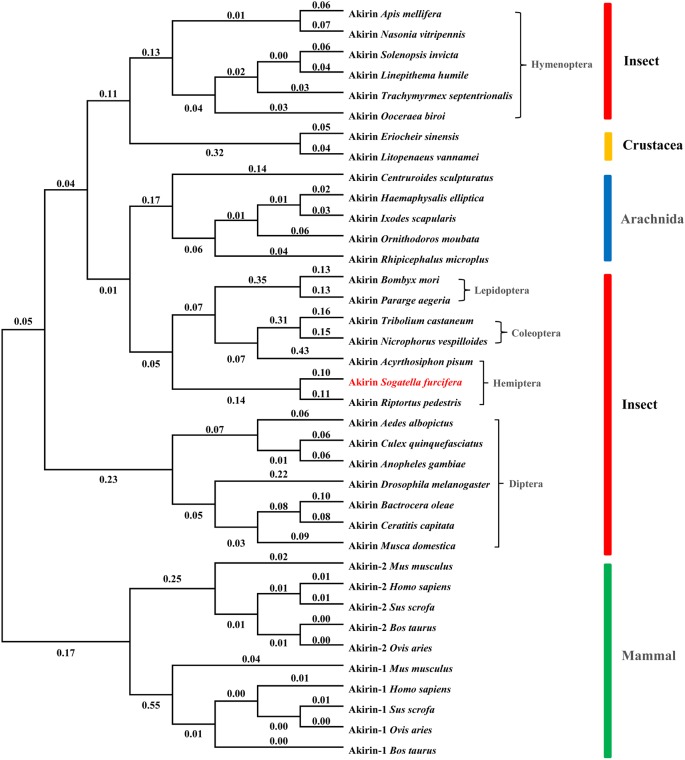
Evolutionary analysis of SfAkirin. The phylogenetic tree was constructed with MEGA 7.0 using the neighbor-joining method. SfAkirin is marked red. The number of branch lengths are shown next to the branches.

The phylogenetic tree was constructed with MEGA 7.0 using the neighbor-joining method. Evolutionary analysis showed that Akirins were divided in two clusters: those of invertebrates (insects, crustaceans, and arachnids) and vertebrates (mammals), and mammalian Akirins were further subdivided into two groups: Akirin-1 and Akirin-2 (**Figure 2**). Interestingly, we found that not all insect Akirins were clustered together but were divided into two branches separated by crustacean and arachnid. Akirins of insects from orders Coleoptera, Hymenoptera, Hemiptera, and Lepidoptera were clustered together, and insects from order Diptera was cluster in another branch. SfAkirin was clustered with the proteins of the first insect group, and was evolutionary closer to Akirins of such relatively distant species as arachnids than to those of some insect orders like Diptera and Hymenoptera. UPGMA method was also used to analyze evolutionary relationship of Akirin (date not shown), and which got the similar result.

### Tissue-Specific Expression of SfAkirin

**FIGURE 3:**
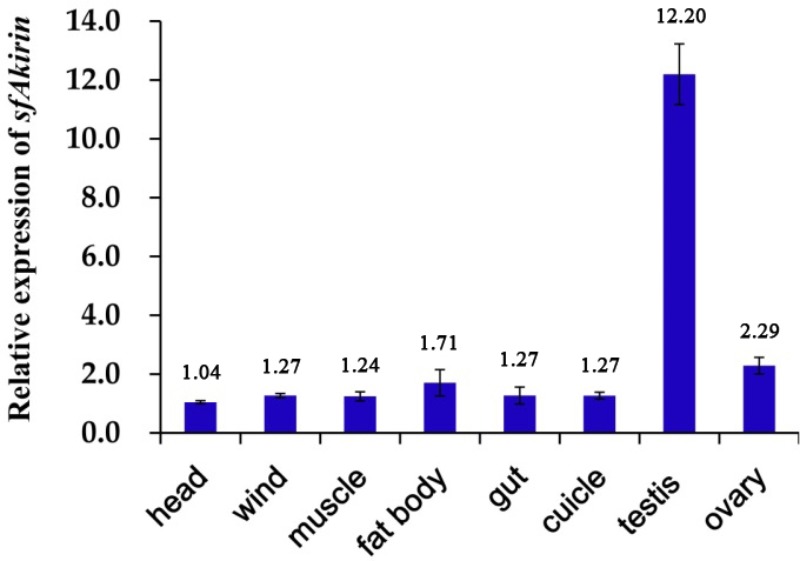
Tissue distribution of *SfAkirin* mRNA. The Actin 1-encoding gene was used as internal control.

The expression profile of the *SfAkirin* gene in *S. furcifera* nymphs was analyzed by qRT-PCR. As shown in **Figure 3**, *SfAkirin* mRNA was detected in all tested tissues (including the head, fat body, gut, wind, cuticle, muscle, testis, and ovary), with the highest expression observed in the testis and the lowest in the head. Thus, the *Akirin* mRNA level in the testis was 11.7 times higher and those in the fat body, gut, wind, cuticle, muscle, and ovary approximately 1.2–2.2 times higher compared to the head (**Figure 3**).

### Gene Expression in *S. furcifera* Challenged With Bacteria

**FIGURE 4:**
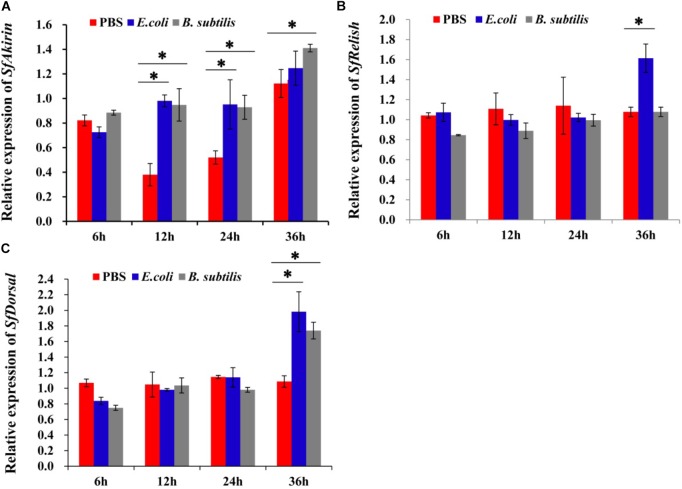
Expression profiles of *SfAkirin, SfRelish*, and *SfDorsal* mRNA in *S. furcifera* challenged with *E. coli* or *B. subtilis*. **(A)** The mRNA expression of SfAkirin post *E. coli* or *B. subtilis* injection; **(B)** The mRNA expression of SfRelish post *E. coli* or *B. subtilis* injection; **(C)** The mRNA expression of SfDorsal post *E. coli* or *B. subtilis* injection. The results are shown as the mean ± SD (*n* = 3). ^∗^Above bars means a statistically significant difference between *E. coli* or *B. subtilis* injection group and control group (*P* < 0.05).

To determine whether SfAkirin has immunity-related functions, *S. furcifera* was injected with heat-inactivated bacteria and analyzed for mRNA expression of *SfAkirin, SfRelish*, and *SfDorsal* by qRT-PCR. As shown in **Figure 4A**, there was no significant difference in *SfAkirin* transcription between control (PBS) and bacteria-injected groups at 6 h or between control and the *E. coli* group at 36 h post challenge, however, *SfAkirin* levels significantly increased at 12 and 24 h after *E. coli* challenge and increased at 12, 24, and 36 h after *B. subtilis* challenge. At the same time, the expression of *SfRelish* was significantly increased by *E. coli* (**Figure 4B**), whereas that of *SfDorsal* – by both *E. coli* and *B. subtilis* (**Figure 4C**) at 36 h post injection.

### *SfAkirin* Silencing Reduced the Transcription of *Relish* and *Dorsal*

**FIGURE 5:**
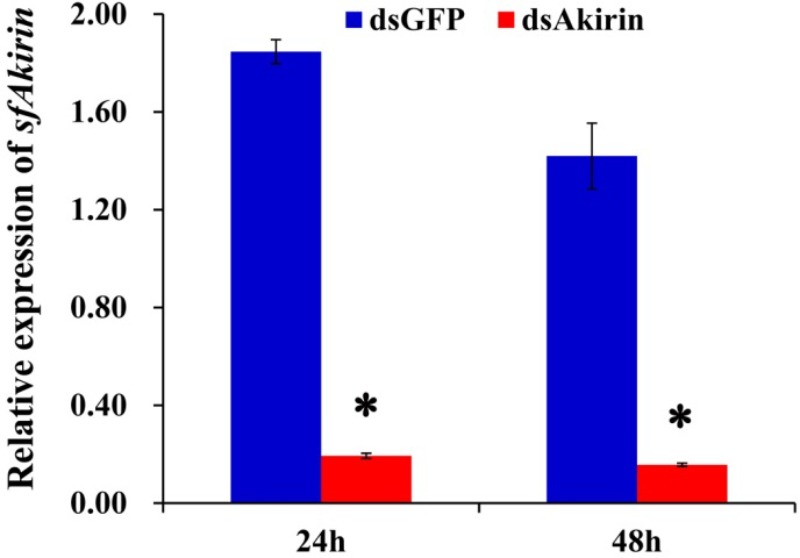
*SfAkirin* mRNA expression in *S. furcifera* injected with dsRNA specific for *Akirin* (dsAkirin) or *GFP* (dsGFP). The results are shown as the mean ± SD (*n* = 3); ^∗^*P* < 0.05 compared with dsGFP.

As shown in **Figure 5**, a significant (10-fold) decrease in *SfAkirin* mRNA expression was observed 24 h after *S. furcifera* injection with *SfAkirin* dsRNA (dsAkirin) compared to the control dsGFP group (*P* < 0.05) and the effect lasted up to 48 h post-injection (*P* < 0.05), indicating efficient silencing of *SfAkirin* expression.

### Silencing *SfAkirin* Reduced the Transcription of *Relish* and *Dorsal*

**FIGURE 6:**
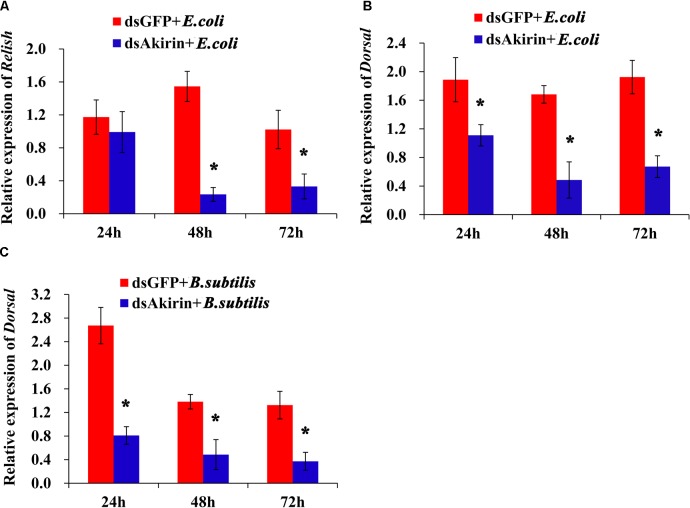
Relative mRNA expression of *Relish* and *Dorsal* in *S. furcifera* with reduced *SfAkirin* expression after bacterial challenge. *S. furcifera* was first injected with dsAkirin or dsGFP and after 24 h treated with *E. coli* or *B. subtilis* and analyzed for mRNA expression at the indicated times. **(A)** *Relish* mRNA levels after *E. coli* injection. **(B)** *Dorsal* mRNA levels after *E. coli* injection. **(C)** *Dorsal* mRNA levels after *B. subtilis* injection. The results are shown as the mean ± SD (*n* = 3); ^∗^Above bars indicate a statistically significant difference (*P* < 0.05).

The mRNA levels of *Relish* and *Dorsal* in *S. furcifera* with normal and reduced SfAkirin expression were examined after challenge with bacteria. *Relish* was significantly downregulated in the dsAkirin + *E. coli* group at 48 and 72 h compared to dsGFP + *E. coli* groups (*P* < 0.05; **Figure 6A**), whereas *Dorsal* was downregulated in the dsAkirin + *E. coli* group at 24, 48, and 72 h compared to the dsGFP + *E. coli* group (*P* < 0.05, **Figure 6B**). Similar results were obtained when *S. furcifera* was challenged with *B. subtilis*: *Dorsal* mRNA was downregulated in the dsAkirin + *B. subtilis* group at 24, 48, and 72 h compared to dsGFP + *B. subtilis* groups (**Figure 6C**).

## Discussion

The NF-κB signaling plays a central role in the innate immunity of insects, presenting the first line of defense against invading pathogens. However, to date, there were no studies on NF-κB signaling in *S. furcifera*. Akirin is a conserved nuclear transcription factor that regulates the NF-κB pathway in innate immune responses (Goto et al., 2008). In *Drosophila*, Akirin controls only the Imd pathway and is not involved in the regulation of the Toll pathway (Goto et al., 2008), which is in contrast to other invertebrates such as *C. rogercresseyi* and *L. vannamei*, where Akirin regulates both Imd and Toll pathways (Carpio et al., 2013; Hou et al., 2013). Although, sequences of the *Akirin* gene from many insect species have been submitted to the GenBank database, research on Akirin functional activity in insects is limited, and relevant studies have only been conducted on *D. melanogaster* and *A. aegypti* (Goto et al., 2008; da Costa et al., 2014).

In the present study, we performed homology analysis using a transcriptome database and identified conserved nucleotide and amino acid sequences in *Akirin* genes of insect species. Our results indicate that Akirins of insects from different orders have a high degree of homology and carry highly conserved amino acid motifs at various regions. In particular, the KRR/QRC sequence at the Akirin N-terminal region (residues 23–27) was determined as an NLS, indicating that the protein functions strictly within the cell nucleus. Phylogenetic analysis of Akirins from invertebrates (including insects, crustaceans, and arachnids) unexpectedly showed that not all insect Akirins were clustered together in the evolutionary tree but were separated into two groups with crustaceans and arachnids positioned in between. SfAkirin was clustered with the proteins of the first insect group, and was evolutionary closer to Akirins of such relatively distant species as arachnids than to those of some insect orders like Diptera and Hymenoptera. These results provide information on the phylogeny and evolution of the *Akirin* gene and suggest that Akirin function may have certain variations in different insects, which should be investigated in further studies on Akirin activity.

Analysis of *Akirin* transcription in different *S. furcifera* tissues indicated that although *Akirin* mRNA was detected in all types of tissues examined, its expression varied significantly: the highest level was observed in the testis, which exceeded that in the head by 11.7 times, and then in the ovary. These results are consistent with those obtained for Pacific white shrimp *L*. *vannamei* (Hou et al., 2013) and suggest a role of Akirin in growth and development of invertebrates, which is supported by previous findings that *Akirin* gene deficiency in *Drosophila* conferred lethality at the embryonic stage (Goto et al., 2008). Furthermore, *Akirin* expression in immunity-related tissues such as the fat body indicates that Akirin can be involved in the immune response of *S. furcifera*.

In *Drosophila*, Akirin acts in parallel with the NF-κB transcription factor downstream of the Imd pathway and was required for defense against gram-negative bacteria (Goto et al., 2008). In our study, *SfAkirin* expression significantly increased after *E. coli* and *B. subtilis* challenge, indicating that Akirin may be involved in early immune responses and is likely to regulate immune sensitivity to both gram-positive and gram-negative bacteria. To further investigate the functional mechanisms underlying Akirin involvement in the NF-κB immune signaling, we silenced *Akirin* expression in *S. furcifera* prior to bacterial challenge, which resulted in reduced expression of the *Relish* and *Dorsal* genes related to the NF-κB-dependent Imd and Toll pathways, respectively. These data suggest that Akirin may control the immune response of *S. furcifera* against bacterial infection via both Imd and Toll signaling pathways, which is in contrast to its role in *Drosophila*, where it is involved only in the Imd pathway (Goto et al., 2008). Our evolutionary analysis shows that SfAkirin is more closely related to *L. vannamei* (shrimp) Akirin than to some insect Akirin, which is consistent with a similarity in immune functions, as *Akirin* silencing in *L. vannamei* significantly reduced the expression of *Dorsal* and *Relish* following challenge with bacteria (Hou et al., 2013).

Previous studies on the regulation of NF-κB immune signaling by Akirin in insect species were performed only on *Drosophila*. Our findings indicate that Akirin exerts more comprehensive regulation of the NF-κB inmmune signaling in insects than previously thought and, thus, should contribute to understanding of Akirin functional activity across different species.

## Conclusion

The results obtained in the present study suggested that SfAkirin is evolutionarily conserved and its expression is significantly increased after *E. coli* and *B. subtilis* challenge. RNAi mediated knockdown of SfAkirin significantly reduced the expression of NF-κB dependent transcription factors, *Dorsal* and *Relish*, post *E. coli*, and *B. subtilis* challenge, respectively. Hence, we proposed that SfAkirin might function as a positive regulator of NF-κB immune signaling in innate immunity of *S. furcifera*.

## Ethics Statement

All applicable international, national, and/or institutional guidelines for the care and use of animals were followed.

## Author Contributions

JC and XJ performed the experiments. X-LX and B-PZ analyzed the data. D-WZ and JC wrote and revised the manuscript.

## Conflict of Interest Statement

The authors declare that the research was conducted in the absence of any commercial or financial relationships that could be construed as a potential conflict of interest.

## Footnotes

1 http://us.expasy.org/tools/

2 http://blast.ncbi.nlm.nih.gov/
